# Detecting multiple simultaneous and sequential feature changes

**DOI:** 10.3389/fcogn.2024.1436351

**Published:** 2024-11-26

**Authors:** Richard D. Wright, Amelia C. Pellaers, Ryan T. deKergommeaux

**Affiliations:** Department of Psychology, Simon Fraser University, Burnaby, BC, Canada

**Keywords:** attention, change detection, flicker task, change magnitude, visual search, sequential presentation

## Abstract

The failure to notice changes to objects is called change blindness, and it is often studied with the flicker task. Observers performing this task see two rapidly alternating but slightly different stimulus displays that are usually photos of real-world scenes. In order to detect the change, they must compare objects in the pre-change scene with objects at the same locations in the post-change scene to determine whether they are the same or different. It has been proposed that change blindness can occur when the memory representation of a pre-change object is incomplete and thereby impairs the same/different comparison with the post-change object at the same location. It has also been proposed that even with intact pre-change object memory representations, failure of same/different comparisons for other reasons can cause change blindness. The goal of the current study was to conduct flicker task experiments to examine both proposals. We conducted the current experiments with non-photographic stimuli, varied the degree of feature-based change of colored lines and found that the greater degree of change, the faster the same/different comparisons, and the faster that changes were detected. We also examined the representation integrity account of change blindness by comparing detection times of target objects that underwent a single feature change with those that underwent multiple sequential feature changes. The latter were detected faster, which suggests that multiple identities of these sequentially changing objects were stored in memory and facilitated change detection. In another experiment we found that objects that underwent multiple sequential feature changes were not detected as fast as those that underwent multiple simultaneous feature changes. This is consistent with the representation account of change blindness and suggests that memories of multiple sequentially changing object identities are transient and may become less complete over time. And more generally that multiple simultaneous and multiple sequential feature-based changes to these stimuli can show the extent to which memory is involved when searching for flicker task targets. The results of the current study indicate that both the comparison failure and the representation integrity proposals can account for change blindness.

## Introduction

Change blindness is the failure to notice sometimes surprisingly large changes to visual scenes, and it can be studied in the laboratory with the flicker task (Rensink, 2018; Rensink et al., 1997; Simons and Rensink, 2005). When participants perform this task, they see a flickering stimulus display that is actually pre-change and post-change stimulus displays that are presented in rapid alternation. Some aspect of the scene is different in the post-change display (e.g., object color or shape or an entire object may be deleted) and participants searching for it press a response key once they detect the change. Finding it, however, often requires a good deal of time because blank screens are briefly presented between each stimulus display to perceptually mask the abruptness of the change so that it does not capture attention. Ten seconds or more is often required to detect the change (e.g., Vanmarke et al., 2017).

There are several hypotheses about the cause of change blindness. Some researchers have proposed that change blindness occurs when observers' initial encoding of a pre-change object's memory representation is too limited to allow them to notice a change (e.g., Noë et al., 2000; O'Regan and Noë, 2001). Others have proposed that the pre-change object's memory representation is initially encoded more completely, but that change blindness occurs because this pre-change object representation is fragile and can be degraded or overwritten or even forgotten (e.g., Beck and Levin, 2003; Becker et al., 2000; Levin et al., 2018, 2002; Tatler, 2001). In both cases, change blindness is said to occur because, when the observer inspects the post-change object, the memory representation of the pre-change object is at best incomplete and, for this reason, the observer's same/different comparison of the pre- and post-change objects is impaired. Another hypothesis is that change blindness can occur even when the memory representation of the pre-change object is sufficiently complete and that change blindness is simply the result of inefficient or failed same/different comparisons of intact pre-change object representations and post-change objects (e.g., Angelone et al., 2003; Hollingworth and Henderson, 2002; Mitroff et al., 2004; Ryan and Cohen, 2004; Scott-Brown et al., 2000; Simons et al., 2002). In summary, one type of hypothesis holds that the occurrence of change blindness is associated with the integrity of pre-change object memory representations. The other type of hypothesis holds that its occurrence may be less dependent on representation integrity and instead is associated with inefficient or failed pre- and post-change object same/different comparisons.

When a pre-change display (A) changes to a post-change display (A′) only once, observers may fail to detect the change and thereby remain change blind. The flicker task, however, involves continuous cycling of displays A and A′ so that the change occurs over and over again until, on most experiment trials, observers eventually detect it and therefore are no longer change blind. Search for flicker task changing objects almost always occurs in a serial manner. That is, a series of same/different comparisons are made between the currently inspected object and a memory representation of the object at the same location in the previously viewed display (e.g., Wolfe et al., 2006). If the object identities are the same, then search continues with comparisons of objects at other locations in displays A and A′ until a mismatch is found. The flicker task is therefore a way to measure the *degree* of change blindness that observers experience when different types of changes occur in visual scenes. Flicker task response times indicate how long it takes before same/different comparisons of pre-change and post-change targets no longer fail. The longer it takes for the comparison to succeed and for observers to detect the changing target, the less efficient the comparison process is.

The speed of search for changes is also affected by the similarity of the objects being compared. The results of one experiment, for example, indicated that the greater the similarity of pre- and post-change novel shape objects, the slower that changes were detected (Williams and Simons, 2000). This is also the case when participants perform target present/absent visual search tasks. That is, the greater the similarity of two objects being compared, the more time is required to determine a mismatch, and the slower the search (e.g., Barras and Kerzel, 2017; Duncan and Humphreys, 1989; Geng and Witkowski, 2019; Treisman and Gormican, 1988; Wolfe et al., 1992; Zhang and Onyper, 2020). Wolfe et al. (1992), for example, found that the more similar the orientation of a target line to that of surrounding non-target lines, the longer it took participants to find the target. And there appeared to be a linear relationship between the degree of similarity of the target and non-targets and search time. These results indicate that if this similarity effect was studied with the flicker task, the greater the difference between pre- and post-change items, the greater the change magnitude and the faster that mismatches would be detected.

Since the publication of Rensink et al. (1997) seminal study in which the flicker task was first used, the effect of pre- and post-change object change magnitude on flicker task detection time has been examined in only a limited way. One reason why is that most flicker task studies in the literature to date involved photographic stimulus displays (e.g., Ortiz-Tudela et al., 2023; Utochkin and Wolfe, 2018). Photographic stimuli are most commonly used, in part, because they are said to be more naturalistic and thereby may increase the face value of the flicker task as a way to study how attention operates in the real world (e.g., Simons and Levin, 2003, p. 313). And so most flicker task experiments have been conducted to study questions such as what effect scene properties (e.g., complexity, gist, and scene inversion) and target object salience (e.g., central vs. marginal interest, priming, expertise, salience to neurodiverse participants) have on change detection efficiency (e.g., Beck et al., 2007; Fletcher-Watson et al., 2012; Hobson et al., 2013; Vanmarke et al., 2017; Zelinsky, 2003). But attempting to study a change magnitude effect on response times by systematically varying the degree of change of an object within a photograph is challenging and can alter the object's relative salience. This is one reason why the flicker task has rarely been used to examine a change magnitude effect involving an individual object. And why some researchers who examined the effect of change magnitude on detection times using photographic stimuli chose instead to vary pictorial scene complexity rather than individual object complexity (e.g., Beck et al., 2007). Varying the change magnitude of individual objects in photographs can be challenging to do with precision.

If non-pictorial stimuli like simple colored lines are used, flicker task experiments can be conducted to study a change magnitude effect on response times in a systematic way because the degree of change of these lines can be varied with precision. One goal of the current study was to determine whether there is a linear relationship between feature-based change magnitude and flicker task target detection time; and also how the change magnitude of these targets may be related to same/different comparison efficiency. The question about what constitutes a feature remains an empirical issue but there is a general consensus among visual search researchers that a feature is a characteristic like color or size or orientation that can guide attention in a bottom-up manner and can lead to rapid object detection (see Wolfe and Horowitz, 2017). Search for a target, for example, can be quite fast if it possesses a unique feature not shared with any other object in the field of view (e.g., the only red one) (e.g., Treisman and Gelade, 1980; Wolfe et al., 1989). And some researchers found that search is even faster when the target possesses multiple unique features not shared with any other object (e.g., color & orientation) as opposed to a single unique feature (e.g., color) (e.g., Krummenacher et al., 2001, 2002; Mordkoff and Yantis, 1993). With this finding in mind, we conducted the current study, in part, to determine whether flicker task search speed is affected by the degree of change of a single target feature and also by the number of target features that changed simultaneously. That is, if the target change involves two features, will it be detected faster than if it involves a single feature? And, similarly, if the change involves three features, will it be detected faster than if it involves only two features or a single feature? A finding that target change magnitude affects flicker task response times in this way would be more consistent with the comparison inefficiency account of change blindness than the object memory representation integrity account.

Another advantage of using non-pictorial stimuli like simple colored lines when conducting flicker task experiments is that they are well suited for multiple sequential target changes. Virtually all flicker task experiments in the current literature involve a single change that occurs back-and-forth as stimulus displays A and A′ are presented in alternation. It is possible, however, to change the identities of a target object in multiple ways while the observer searches for it. The color of a target line in one display, for example, could be different in a post-change display; and, before it is found, its size could be different in the next display; and then, before the target is found, its orientation could be different in the next display, and so on. Another goal of the current study was to determine the extent to which sequential presentation of different versions of the target at a particular location would result in the creation of multiple memory representations that may be involved in same/different target identity comparisons. We examined this by comparing the time observers required to detect targets with single and with multiple sequential feature changes. If the latter are detected faster, then this suggests that search for a multiple sequential change target involves a same/different comparison of the currently viewed item and memory representations of several previous item identities. And that flicker task same/different comparison efficiency is facilitated when several memory representations of different target identities are involved.

When a flicker task target changes sequentially in multiple ways, it may be the case that the memory representation of the immediately previous target identity may be more complete than the representations of target identities that were presented earlier in the sequence. Although this has yet to be studied with the flicker task, the effect of multiple sequential stimulus changes on memory accuracy has been studied with the one-shot task (see e.g., Bharti et al., 2020; Schneegans et al., 2023). This task is so named because participants are only allowed one view of a pre-change display (i.e., one shot) and then must store information about all items in this display in visual working memory (e.g., Luck and Vogel, 1997). The primary dependent measure is how accurately participants can determine whether pre- and post-change displays are the same or different. It is however a memory task and does not provide as much information as the flicker task about factors that influence change blindness and serial search efficiency (cf. Pailian et al., 2020). The results of experiments involving a variant of the one-shot task in which small sets of items were presented sequentially vs. simultaneously showed that memory accuracy is poorer for sequentially presented items than for simultaneously presented items, and particularly for those at earlier positions in the sequence (e.g., Allen et al., 2006, 2014; Brown et al., 2017; Gorgoraptis et al., 2011; Jaswal and Logie, 2011). In other words, there is a recency effect whereby memory for features of the most recently presented item is more accurate than memory for features of the second most recently presented item, and so on. Some researchers have proposed that this occurs because memory representations of items at earlier positions in the sequence are vulnerable to interference by subsequent items, and particularly when sequentially presented items appear at the same location (e.g., Hitch et al., 2020; Kool et al., 2014; Pertzov and Husain, 2014; Schneegans et al., 2021; Treisman and Zhang, 2006). That is, the more recent that an item is presented in the sequence, the more complete its memory representation is said to be. If a flicker task experiment was conducted with multiple sequential change targets, then it may be the case that memory representations of targets presented earlier in the sequence would contain only partial information about their identities.

As mentioned previously, if flicker task response times are faster for multiple sequential change targets than for single change targets, then this suggests that same/different comparison efficiency is facilitated when several memory representations of different target identities are involved. But it also may be the case that memory representations of the targets presented earlier in the sequence contain only partial information about their identities. We examined this in the current study by comparing the speed of search for targets with three *sequential* feature changes across successive flickers with that for targets with all three of these feature changes occurring *simultaneously*. If triple feature simultaneous change target detection time is faster, then this may be because all three feature differences are apparent in a single memory representation of the immediately previous version of the target. Slower triple feature sequential change target detection time, on the other hand, may indicate that feature difference information in memory representations of targets presented earlier in the sequence may have degraded. In other words, while target identity information in earlier memory representations may have some effect on same/different comparison efficiency, this information may be only *partially* complete. This would be consistent with the representation integrity hypothesis about the cause of change blindness.

In summary, we conducted flicker task experiments to determine the extent to which change blindness would be accounted for by comparison failure or by the integrity of pre-change object memory representations. The stimuli were simple colored lines that, we argue, are better suited than photographs for systematic and precise variation of target change magnitude, and for sequentially changing target identity in multiple ways across flickers. Experiments 1 and 3 were conducted to examine how the result of varying target change magnitude may be consistent with the comparison account, and that Experiment 4 was conducted to determine the extent to which memory representations of sequentially presented targets would degrade and therefore be consistent with the representational integrity account.

## Experiment 1

**Figure 1:**
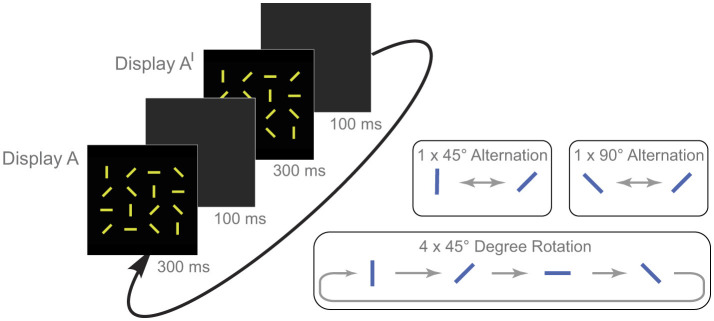
An example of a flickering stimulus display in Experiment 1 and the three types of targets. As display A and A′ were presented in alternation, participants searched for the line that changed orientation. In this figure, the target line is in the top row in the far right column. The 1 × 45° and the 1 × 90° targets alternated back and forth with 45° and 90° orientation change magnitudes respectively. And the 4 × 45° target line rotated clockwise by 45° increments across a series of four flickers.

The first experiment was conducted to examine how varying the degree of change of a single feature of flicker task targets would affect change detection response times. Stimuli were small lines and the target change involved variation of line orientation. On some trials, a single change occurred as the orientation of the target line alternated back and forth. On other trials, a multiple sequential change occurred that involved four different line orientations (see Figure 1). We expected that the greater the degree of target line orientation change, the faster that targets would be detected. If so, then this would indicate that varying the change magnitude of the target affects the efficiency of same/different flicker task comparisons. The experiment was also an initial attempt to determine whether search for multiple sequential change targets would be faster than search for single change targets.

### Method

#### Participants

Forty-four Simon Fraser University (SFU) undergraduate students were recruited through the Psychology department subject pool. Their ages ranged from 17 to 23 years (26 females & 18 males) and they received course credits for their participation. All participants had normal or corrected-to-normal vision, normal color vision (tested with Ishihara isochromatic plates), and no history of seizure.

#### Apparatus

All experiments in this study were carried out with PC computers and 19^′′^ LCD monitors (1,240 × 1,028 px display resolution). The experiments were controlled by and responses were recorded using E-prime 2.0 software. Participants' viewing distance in each experiment was 60 cm from the monitor.

#### Stimuli

Stimulus items on each trial were 16 yellow lines (rgb 217, 224, 33) presented on a black background (rgb 0, 0, 0). Each line subtended 4 × 1° of visual angle and was oriented either vertically, horizontally, at an angle from upper-right to lower-left, or at an angle from upper-left to lower-right. The lines were arranged in a 4 × 4 array that subtended 28 × 28° of visual angle. On each trial, there were an equal number of lines of each orientation. As seen in Figure 1, display A and A′ were presented for 300 ms, and the intervening blank gray screens (rgb 75, 75, 75) were presented for 100 ms. When the stimulus displays were presented in alternation, the orientation of one of the lines (the target) changed across flickers. The three types of targets were a line that changed orientation back and forth by 90° across flickers (1 × 90°); a line that changed orientation back and forth by 45° across flickers (1 × 45°); and a line that changed orientation by 45°, but in four clockwise increments (4 × 45°). On those trials, there were four different stimulus displays (A, A′, A″ & A^‴^).

#### Procedure

Participants were instructed to find, as quickly as possible, the target line that was changing. When they detected it, they pressed a response-box button that stopped the display flickering and the reaction timer. At that point, one of the stimulus displays remained on the screen and a mouse cursor arrow became visible. To confirm that they had accurately located the target, participants moved the arrow and clicked on the line that they believed had been changing. If they clicked on a line that had not changed orientation, then this was recorded as a change-localization error (a rare occurrence). And if they did not respond within 60 seconds (also a rare occurrence), then the trial was terminated. These null responses were recorded as time-out errors. After the response (or a trial time-out), there was a 1-second interval before the beginning of the next trial. Participants completed a block of five practice trials and then, over the course of a 30-min testing session, completed three blocks of 48 randomly ordered data trials with 60-second rest periods between blocks.

### Results

**Figure 2:**
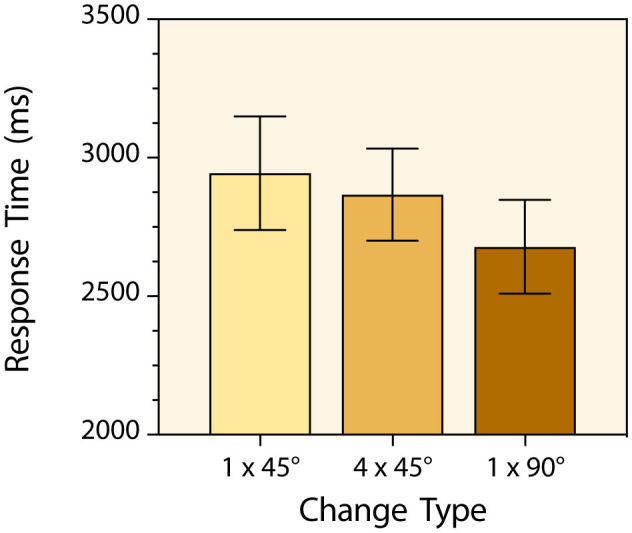
Mean change detection response times for the three types of targets in Experiment 1. The 1 × 45° and 1 × 90° labels refer to back-and-forth line orientation changes of 45° and 90° respectively. The 4 × 45° label refers to four sequential clockwise line orientation changes of 45°. Error bars indicate 95% confidence intervals.

Participants made very few time-out and change-localization errors (97.3% response accuracy). No speed-accuracy trade-off occurred, and no further analysis was carried out with the response accuracy data. Prior to the response time analysis, a few trials (2.3%) with response times ± 3 standard deviations away from the corresponding trial-type mean were excluded as outliers. A one-way repeated measures analysis of variance with change-type serving as the within-subjects factor was then conducted with the mean response times for each participant in each condition and there was a main effect [*F*_(2, 86)_ = 12.07, *p* < 0.0001, $\eta_{p}^{2}$ = 0.219] (Figure 2). *Post hoc* tests with Bonferroni correction indicated that participants detected 1 × 90° changes significantly faster than 1 × 45° changes (*M* = 2,678 ms & *M* = 2,944 ms respectively) [*t*_(43)_ = 5.40, *p* < 0.0001, *d* = 0.81]. They also detected 1 × 90° changes significantly faster than the sequence of four 45° rotational changes (*M* = 2,678 ms & *M* = 2,867 ms respectively) [*t*_(43)_ = 3.33, *p* = 0.005, *d* = 0.50]. They did not, however, detect the sequence of four 45° rotational changes significantly faster than single (back & forth) 1 × 45° changes (*M* = 2,867 ms & *M* = 2,944 ms respectively) [*t*_(43)_ = 1.28, *p* = 0.635, *d* = 0.19].

### Discussion

These results indicate that the less similar the orientations of target lines in pre- and post-change displays, the sooner that participants detected the mismatch, and the faster the response time. As expected, the larger 90° orientation change was detected faster than the 45° orientation change. This change magnitude finding is consistent with the comparison account of change blindness more so than the representation account. There was no significant difference, however, between mean single change (1 × 45°) and mean multiple sequential change (4 × 45°) target detection times. Response times were quite rapid, however, and this may be because the stimulus displays were somewhat homogeneous. The color and size of the stimulus lines were the same and only their orientation differed. Perhaps if the displays were more heterogeneous and stimuli varied across several feature dimensions like color, orientation, and size, then response times would not be quite as fast and a significant difference between the single and multiple sequential target response times would be more likely.

## Experiment 2

This experiment was another attempt to determine whether search for multiple sequential change targets would be faster than search for single change targets. There was no significant difference between the mean response times for detecting multiple sequential (4 × 45°) and single orientation change (1 × 45°) targets in Experiment 1 with stimuli that were all the same size and color. But in this experiment, the stimuli varied in color as well as orientation, which made the displays more heterogeneous than those of Experiment 1. We expected that with this greater display heterogeneity, multiple sequential orientation change targets would be detected faster than single orientation change targets, and that multiple sequential color change targets would be detected faster than single color change targets.

### Method

#### Participants

Thirty-five SFU undergraduate students were recruited through the Psychology department subject pool. Their ages ranged from 17 to 24 years (29 females & 6 males) and they received course credits for their participation. All participants had normal or corrected-to-normal vision, normal color vision (tested with Ishihara isochromatic plates), and no history of seizure.

#### Stimuli and procedure

**Figure 3:**
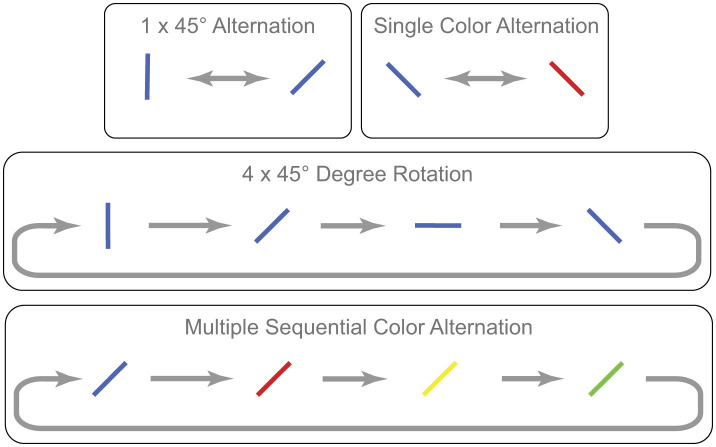
Examples of the four types of targets in Experiment 2.

Stimulus items on each trial were 16 lines that varied in color and orientation. Stimulus displays were therefore more heterogeneous than those in Experiment 1. The four colors were red (rgb 255, 0, 0), green (rgb 0, 255, 0), blue (rgb 0, 83, 255), yellow (rgb 255, 176, 0), and the items were presented on a black background (rgb 0, 0, 0). Each line subtended 4 × 1°of visual angle. As in the first experiment, lines were oriented either vertically, horizontally, at an angle from upper-right to lower-left, or at an angle from upper-left to lower-right. And they were again arranged in a 4 × 4 array that subtended 28 × 28° of visual angle. Two of the target changes involved orientation and two involved color (Figure 3). In particular, one involved a single back-and-forth 45° change in orientation of a line that remained the same color. One involved a sequence of four 45° clockwise increments of a line that remained the same color. Another involved a single back-and-forth color change of a line that remained at the same orientation (e.g., red ↔ green). And another involved a sequence of four different color changes that cycled continuously while the line remained at the same orientation (e.g., red ⇒ green ⇒ blue ⇒ yellow ⇒ red, etc.). Participants in this experiment performed the same type of flicker task as in the previous experiment. After a block of five practice trials, they completed four blocks of 48 randomly ordered data trials with 60-second rest periods between blocks.

### Results

**Figure 4:**
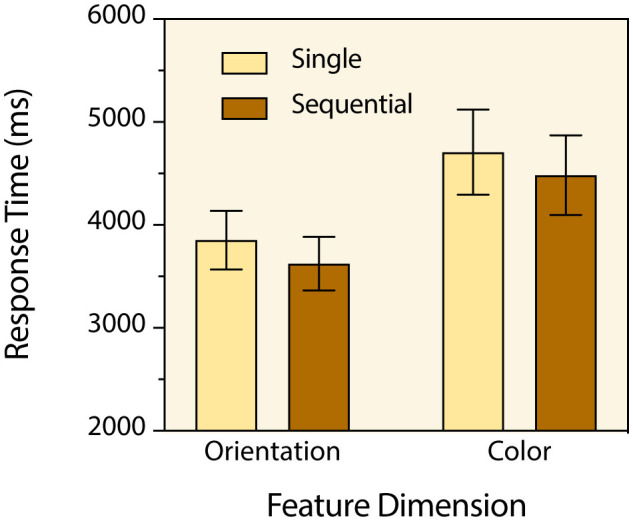
Mean change detection response times for the four types of targets in Experiment 2. Error bars indicate 95% confidence intervals.

As was the case in the previous experiment, participants made very few time-out and change-localization errors (99.1% response accuracy). No speed-accuracy trade-off occurred, and no further analysis was carried out with the response accuracy data. Prior to analysis of response times, a few trials (1.6%) with response times ± 3 standard deviations away from the corresponding trial-type mean were excluded as outliers. A two-way repeated measures analysis of variance with feature change type and single/sequential change type serving as within-subjects factors was then conducted with the mean response times for each participant in each condition. There was a main effect of feature change type [*F*_(1, 34)_ = 39.91, *p* < 0.0001, $\eta_{p}^{2}$ = 0.540] (Figure 4). Orientation changes were detected faster than color changes. There was also a main effect of single/sequential change type [*F*_(1, 34)_ = 30.82, *p* < 0.0001, $\eta_{p}^{2}$ = 0.475]. Unlike in Experiment 1, sequential changes were detected faster than single changes. *Post hoc* tests with Bonferroni correction indicated that participants detected multiple sequential orientation changes significantly faster than single orientation changes (*M* = 3,626 ms & *M* = 3,854 ms respectively) [*t*_(34)_ = 3.0, *p* = 0.0099, *d* = 0.51], and they detected multiple sequential color changes significantly faster than single color changes (*M* = 4,485 ms & *M* = 4,708 ms respectively) [*t*_(34)_ = 2.94, *p* ≤ 0.012, *d* = 0.50].

### Discussion

The results of this experiment show that for both types of features, multiple sequential change targets were detected significantly faster than single change targets. This suggests that participants' same/different comparisons of multiple sequential change targets may have been influenced by more than one memory representation of the target's multiple identities. That is, when searching for these targets, participants may have made a same/different comparison of the currently inspected item's identity with the memory representation of the item's identity at the same location in the immediately previous display as well as memory representations of item identities at the same location in other displays besides the immediately previous one. Although it cannot be assumed that flicker task orientation changes will always be detected faster than color changes, that should be the case in this study because the same four colors and the same line orientation changes (45° or 90°) were used in each of the current experiments.

## Experiment 3

The results of the first experiment showed that the greater the degree of change of a single feature value, the faster that flicker task targets were detected. Experiment 3 was conducted to examine how varying the number of feature dimensions (orientation, color, and size) that changed simultaneously would affect target detection response times. As mentioned in the Introduction section, the results of previous visual search experiments showed that pop out targets with two unique features (color & orientation) were found even faster than pop out targets with a single unique feature (only color) (e.g., Krummenacher et al., 2001, 2002; Mordkoff and Yantis, 1993). In the current experiment, we measured change detection response times for single feature change, double feature change, and triple feature change targets. And we expected that the greater the number of feature dimensions involved when targets changed, the faster that those targets would be detected. This would indicate that flicker task comparison failure does not persist as long when target changes involve multiple features.

### Method

#### Participants

Seventy-nine SFU undergraduate students were recruited through the Psychology department subject pool. Their ages ranged from 17 to 28 years (45 females & 34 males) and they received course credits for their participation. All participants had normal or corrected-to-normal vision, normal color vision (tested with Ishihara isochromatic plates), and no history of seizure.

#### Stimuli and procedure

**Figure 5:**
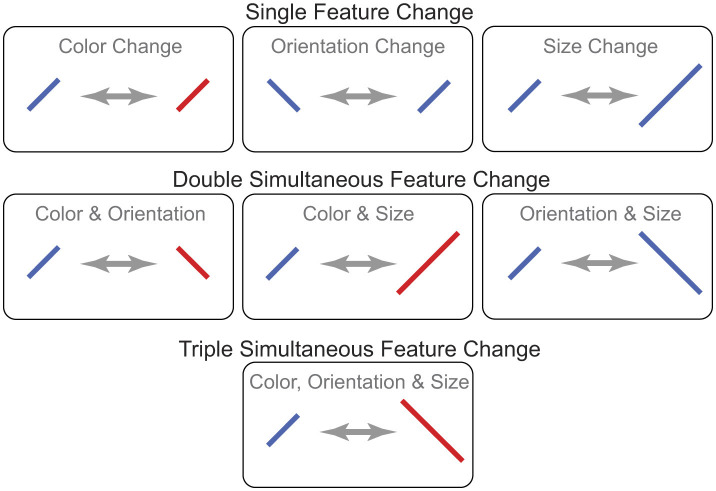
Examples of the three types of targets in Experiment 3. Single feature changes involved only one feature dimension (color or orientation or size). Double simultaneous feature changes involved two of these feature dimensions, and triple simultaneous feature changes involved all three feature dimensions.

Stimulus items on each trial were 16 lines that varied in color, size, and orientation. The four colors were the same as those in the previous experiment and the items were presented on a black background (rgb 0, 0, 0). Each line subtended 2.7 × 1° (small size) or 5.4 × 1° (large size) of visual angle. As in the previous experiments, lines were oriented either vertically, horizontally, at an angle from upper-right to lower-left, or at an angle from upper-left to lower-right. And they were again arranged in a 4 × 4 array that subtended 28 × 28° of visual angle. On some trials, when display A and A′ were presented in alternation, only a single target feature changed (either color, size, or orientation). These were the *single* feature change targets (see Figure 5). Whenever the change involved orientation, the target line angle changed back and forth with a magnitude 90°. On other trials, two of the three types of target features changed at the same time. These were the *double-simultaneous* feature change targets. And on the third type of trial, all three types of target features changed simultaneously. These were the *triple-simultaneous* feature change targets. Participants in this experiment performed the same type of flicker task as in the previous experiment. After a block of five practice trials, they completed three blocks of 48 randomly ordered data trials with 60-second rest periods between blocks.

### Results

**Figure 6:**
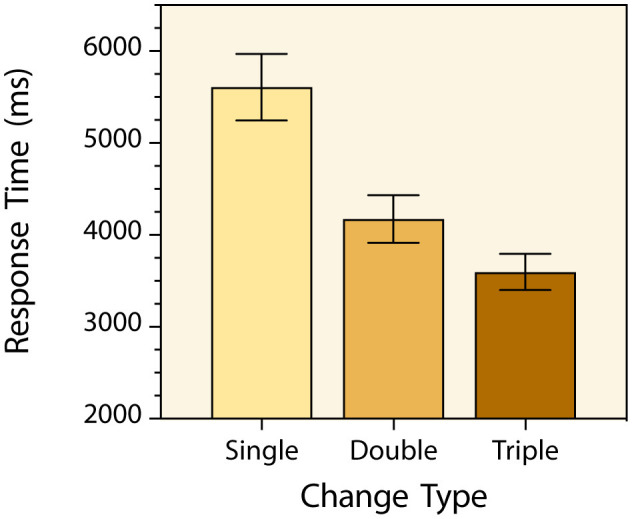
Mean change detection response times for the three types of targets in Experiment 3. The Single, Double, and Triple labels refer to single-feature change, double-feature change, and triple-feature change targets respectively. Error bars indicate 95% confidence intervals.

As was the case in the previous experiments, participants made very few time-out and change-localization errors (97.6% response accuracy). No speed-accuracy trade-off occurred, and no further analysis was carried out with the response accuracy data. Prior to analysis of response times, a few trials (1.7%) with response times ± 3 standard deviations away from the corresponding trial-type mean were excluded as outliers. A one-way repeated measures analysis of variance with change-type serving as the within-subjects factor was then conducted with the mean response times for each participant in each condition and there was a main effect [*F*_(2, 156)_ = 236.7, *p* < 0.0001, $\eta_{p}^{2}$ = 0.752] (Figure 6). The greater the target feature change magnitude, the faster that targets were detected. *Post hoc* tests with Bonferroni correction indicated that participants detected triple-simultaneous feature changes significantly faster than single feature changes (*M* = 3,597 ms & *M* = 5,608 ms respectively) [*t*_(78)_ = 16.65, *p* < 0.0001, *d* = 1.87]. They also detected triple-simultaneous feature changes significantly faster than double-simultaneous feature changes (*M* = 3,597 ms & *M* = 4,174 ms respectively) [*t*_(78)_ = 9.07, *p* < 0.0001, *d* = 1.02]. And they detected than double-simultaneous feature changes significantly faster than single feature changes (*M* = 4,174 ms & *M* = 5,608 ms respectively) [*t*_(78)_ = 15.52, *p* < 0.0001, *d* = 1.75]. As in the first experiment, the less similar the two versions of the target being compared across flickers in the pre- and post-change displays, the faster the mismatch was detected. And the effect size of this linear trend was large [*F*_(1, 156)_ = 446.4, *p* < 0.0001, $\eta_{p}^{2}$ = 0.741].

### Discussion

The results of this experiment show that the greater the number of feature dimensions involved when targets changed, the faster they were detected. And there was a linear relationship between the degree of change and mean response times. Note that the mean single-change response time was slower than that in Experiment 1. This is because single changes in the first experiment involved only orientation changes (detected faster) whereas in this experiment, in addition to orientation, single changes also involved color and size which take longer to detect (see Experiment 2). The results are consistent with the comparison account of change blindness more so than the representation account.

## Experiment 4

The fourth experiment was conducted to determine whether three-feature sequential change targets would be detected faster than single change targets; and also whether targets with three features that changed *simultaneously* would be detected faster than targets with three features that changed *sequentially*. To elaborate, with three-feature sequential change targets, same/different comparisons might involve different target identities in memory representations of displays presented prior to the previously viewed display, as appeared to be the case in Experiment 2. And as suggested previously, this could increase comparison efficiency and enable faster detection of the target identity mismatches than would be the case with single change targets. But it also may be the case that when the three feature changes occur sequentially across flickers, comparison efficiency would not be as great as when the three features change simultaneously across a single flicker. More specifically, when three feature changes occur sequentially across flickers, memory representations of the targets presented earlier in the sequence may contain only partial information about their identities. And this may reduce same/different comparison efficiency relative to three-feature simultaneous change targets because all three feature differences of the latter would be apparent in a single memory representation of the immediately previous version of the target. In other words, information about the three feature changes in memory representations of targets presented earlier in the sequence may have degraded and these representations may be only *partially* complete. If so, then this result would indicate that flicker task comparison efficiency would be influenced by the integrity of memory representations.

### Method

#### Participants

Thirty-six SFU undergraduate students were recruited through the Psychology Department subject pool. Their ages ranged from 18 to 23 years (13 females & 23 males) and they received course credits in exchange for their participation. All participants had normal or corrected-to-normal vision, normal color vision (tested with Ishihara isochromatic plates), and no history of seizure.

#### Stimuli and procedure

**Figure 7:**
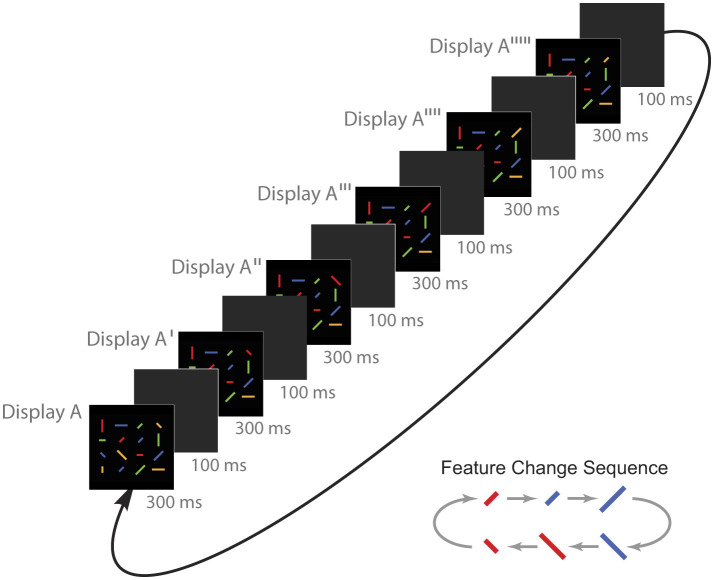
An example of a flickering stimulus display on triple-sequential target trials in Experiment 4. Participants searched for the changing line while they viewed sequential presentations of display A to A′ to A″ to A^‴^ to A^′*′′′*^ to A^′*′′′′*^ to A, and so on. A color change was always followed by a size change, which was always followed by a 90° line orientation change, which was always followed by a color change, and so on in this order. On trials with triple-sequential feature change targets, only one change occurred per flicker. In this example, the target is the line in the top row and in the far right column.

Stimulus items on each trial were 16 lines with the same colors, sizes, and orientations as those in the previous experiment, and they were arranged in the same 4 × 4 array as in the previous experiments. On some trials, only a single target feature changed (either color, size, or orientation). These were the *single* feature change targets. On other trials, all three types of target features changed simultaneously. In the results section, we refer to these as *triple-simultaneous* feature change targets. Both types of targets were also used in Experiment 3 (see Figure 7). On a third type of trial, the three types of target features changed one at a time across a sequence of six flickers. As seen in Figure 7, on this type of trial, a target color change was always followed by a size change, which was followed by a 90° orientation change, which was followed by the target changing back to its original color, and so on. The order of feature changes was as follows: color_(original)_ ⇒ color_(new)_ ⇒ size_(new)_ ⇒ orientation_(new)_ ⇒ color_(original)_ ⇒ size_(original)_ ⇒ orientation_(original)_ ⇒ color_(new)_, etc. In the results section, we refer to these as *triple-sequential* feature change targets. Participants performed the same type of flicker task as in the previous experiments. After completing a block of five practice trials, participants completed three blocks of 48 randomly ordered data trials with 60-second rest periods between blocks.

### Results

**Figure 8:**
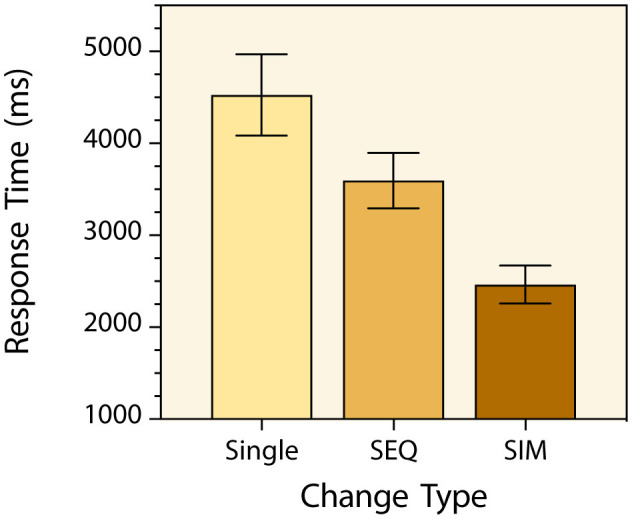
Mean change detection response times for the three types of targets in Experiment 4. The Single, SEQ and SIM labels refer to single-feature change, triple-feature sequential change, and triple-feature simultaneous change targets respectively. Error bars indicate 95% confidence intervals.

As was the case in the previous experiments, participants made very few time-out and change-localization errors (97.5% response accuracy). No speed-accuracy trade-off occurred, and no further analysis was carried out with the response accuracy data. Prior to analysis of response times, a few trials (2.1%) with response times ± 3 standard deviations away from the corresponding trial-type mean were excluded as outliers. A one-way repeated measures analysis of variance with change-type serving as the within-subjects factor was then conducted with the mean response times for each participant in each condition and there was a main effect [*F*_(2, 70)_ = 106.6, *p* < 0.0001, $\eta_{p}^{2}$ = 0.753] (Figure 8). *Post hoc* tests with Bonferroni correction indicated that participants detected triple-simultaneous feature changes significantly faster than single feature changes (*M* = 2,465 ms & *M* = 4,527 ms respectively) [*t*_(35)_ = 11.91, *p* < 0.0001, *d* = 1.99]. They also detected triple-simultaneous feature changes significantly faster than triple-sequential feature changes (*M* = 2,465 ms & *M* = 3,596 ms respectively) [*t*_(35)_ = 10.47, *p* < 0.0001, *d* = 1.75]. And participants detected triple-sequential feature changes significantly faster than single feature changes (*M* = 3,596 ms & *M* = 4,527 ms respectively) [*t*_(35)_ = 6.87, *p* < 0.0001, *d* = 1.15]. As in Experiment 3, the effect size of this linear trend was large [*F*_(1, 70)_ = 212.6, *p* < 0.0001, $\eta_{p}^{2}$ = 0.752].

### Discussion

The results of this experiment show that triple-sequential feature change targets were detected faster than single feature change targets. Like the results of Experiment 2, this indicates that the efficiency of same/different comparisons can be influenced by more than one memory representation of the target. In particular, when searching for a single change target, participants make a same/different comparison that involves the currently viewed object and the identity of the same-location object in a memory representation of the previous display. But when searching for a multiple sequential change target, perhaps participants respond faster because they make a same/different comparisons that involve the identity of the currently viewed object, the identity of the same-location object in the immediately previous display, and also the identities of same-location objects in memory representations of other displays besides the immediately previous one. In addition, flicker task targets were detected faster when three features were changed simultaneously rather than sequentially. There was a greater degree of feature change across a single flicker with the simultaneous change targets than with the sequential change targets, which may have led to faster mismatch detection. Thus, the results of Experiment 4 show that the flicker task with multiple sequential change targets can be used to study the extent to which the completeness of memory representations affects change detection. And they suggest that feature information in memory representations of targets presented earlier in the sequence may have been only partially complete. This is consistent with the representation integrity hypothesis about the cause of change blindness.

## General discussion

This study was conducted with non-photographic stimuli to determine the extent to which flicker task change blindness is caused by comparison failure or by the integrity of pre-change object memory representations. The experiments were carried out to examine how the magnitude of simultaneous and sequential feature-based changes affects the speed of detection of flicker task targets and the results showed that the greater the feature similarity of pre- and post-change target identities, the slower that changes were detected. This indicates that the greater their similarity, the less efficient the same/different comparison processes were. In addition, multiple sequential change targets (one change per flicker) were detected faster than single change targets but not as fast as multiple simultaneous change targets. This indicates that when searching for multiple sequential change targets, participants made same/different comparisons that involved the identity of the currently inspected item (display A), a memory representation of the identity of the item at the same location in immediately previous display (display A′), and also, to some extent, memory representations of identities of items at that location in displays presented prior to the immediately previous display (e.g., display A″ & A^‴^). Slower detection of multiple-feature sequential change targets than multiple-feature simultaneous change targets indicates, however, that feature information in memory representations of targets presented earlier in the sequence may have degraded relative to feature information in memory representations of simultaneous change targets. In other words, the memory representations of targets presented earlier in the sequence may have been only *partially* complete. These findings indicate that flicker task change blindness in the experiments was consistent with both the comparison failure and the representation integrity accounts.

The results of this study show that when flicker task experiments are conducted with simple colored line stimuli, the degree of change can be varied in a systematic and precise way to study the effect of target change magnitude on change detection time. As mentioned previously, the flicker task has rarely been used to study the change magnitude effect because most studies have involved photographic stimulus displays and it can be challenging to systematically vary the degree of change of an object within a photo without also altering its relative salience within the scene. It can be done with precision, however, if non-photographic stimuli like the colored lines in the current experiments are used. The degree to which target features like size and orientation are changed can be, if required, quite small and exact (e.g., a change of only 1°). This allowed us to find that the relationship between pre- and post-change target similarity and speed of its detection is linear. Unlike flicker task studies involving scene-based changes within photos, non-photographic stimuli can be used to precisely vary target feature properties and examine questions more closely related to proposals about feature-based models of visual search (e.g., Treisman, 1998; Wolfe, 2021).

The flicker task is an effective way to examine the effect of multiple sequential target changes on target detection time and the extent to which memory representations of target identities can degrade. This cannot be studied with visual search tasks with static (non-flickering) displays or with one-shot tasks with a single briefly presented pre-change display. But as mentioned previously, the results of some studies using a variant of the one-shot task involving a sequence of four or more different displays showed that memory for features of the most recently presented object is more accurate than memory for features of the second most recently presented object, and so on (e.g., Allen et al., 2006, 2014; Brown et al., 2017; Gorgoraptis et al., 2011; Jaswal and Logie, 2011). And the memory representations of the more recently presented objects are said to be more complete (e.g., Hitch et al., 2020). Hitch et al. (2020, p. 285) proposed that the recency benefit for items presented later in a sequence may be related to the completeness of each item's object file. An object file is a temporary memory representation of an attended item's visual features, spatial location, and temporal position (Kahneman et al., 1992). And this information is said to be fragile and degrade over time (e.g., Wheeler and Treisman, 2002). Hitch et al. hypothesized that visual working memory for sequentially presented items involves cycles in which focused attention creates new and complete object files. And each cycle of object file creation pushes back, in the working memory store, the object files of previously presented items in a way that results in a *recency gradient*. That is, the more recently that an item is presented, the more complete the information in its object file is said to be. But as subsequent items are presented, the object files of earlier presented items are said to be pushed back in the working memory store, and interference by more recently presented items can cause earlier item object file information to degrade and be less complete. Thus, according to this recency gradient hypothesis, information about some sequentially presented items besides the most recent one may still be available in working memory; but the earlier that these items were presented in the sequence, the less information about them may be available because their object file information may have degraded.

The recency gradient hypothesis about memory representation completeness is consistent with the Experiment 4 finding that multiple simultaneous change targets were detected faster than multiple sequential change targets. That is, when participants searched for three-feature sequential change targets, same/different comparisons appeared to involve the target's identity in the currently viewed display and perhaps several alternate target identities in the memory representations of previous sequentially presented displays. But the representations of the less recent versions of the target may have degraded and become less complete as subsequent versions of the target were presented at the same location. When participants searched for three-feature simultaneous change targets on the other hand, same/different comparisons involved only the target's identity in the currently viewed display and the alternate target identity in a memory representation of the previous display. And these comparisons would therefore not involve multiple degraded memory representations of other target identities as may be the case when searching for three-feature sequential change targets. However, the availability of memory representations of alternate identities of multiple sequential change targets, even if partially degraded and not fully complete, may account for the faster detection of three-feature sequential change targets than single feature change targets. This indicates flicker task change blindness can be affected by the integrity of the memory representations involved in the comparison process.

Note that the Experiment 2 results do not indicate which types of feature changes (color, size, orientation, etc.) will be detected fastest when observers perform flicker tasks. Orientation changes were detected faster than color changes in that experiment, but this will not be the case in all flicker task experiments. Change detection speed is affected by the similarity of pre- and post-change objects. And an orientation change would therefore be slower to detect if pre- and post-change object similarity was greater. For example, a target line that changes orientation back and forth by just 5° across flickers would be slower to detect than one that changes orientation by 45°. Similarly, a target line that undergoes a small degree of color change across flickers (e.g., lighter red ↔ darker red) would be slower to detect than one that undergoes a larger degree of color change (e.g., red ↔ green). In other words, depending on the similarity of pre- and post-change targets, color changes in other experiments may be detected faster than orientation changes. And strong conclusions about which types of feature changes are more easily detectable should be made with caution. With any type of feature change, some pre- and post-change targets may be so similar that target detection could take a long time.

In summary, the current results may lead to a better understanding of the extent to which memory representations are involved in flicker task change detection. They are consistent with both the representation integrity and the comparison hypotheses about the causes of change blindness (e.g., Simons and Ambinder, 2005, p. 47; Varakin et al., 2007, p. 746). And they are a step toward closer integration of the flicker task literature, the working memory literature, and the visual search literature.

## Data Availability

The datasets in this study can be found in the OSF online repository and are available at https://osf.io/hpxqv.
